# Embedded UV Sensors in CMOS SOI Technology

**DOI:** 10.3390/s22030712

**Published:** 2022-01-18

**Authors:** Michael Yampolsky, Evgeny Pikhay, Yakov Roizin

**Affiliations:** Tower Semiconductor, Migdal Haemek 2310502, Israel; evgenypi@towersemi.com (E.P.); yakovro@towersemi.com (Y.R.)

**Keywords:** UV sensors, SOI, sterilization, CMOS

## Abstract

We report on ultraviolet (UV) sensors employing high voltage PIN lateral photodiode strings integrated into the production RF SOI (silicon on isolator) CMOS platform. The sensors were optimized for applications that require measurements of short wavelength ultraviolet (UVC) radiation under strong visible and near-infrared lights, such as UV used for sterilization purposes, e.g., COVID-19 disinfection. Responsivity above 0.1 A/W in the UVC range was achieved, and improved blindness to visible and infrared (IR) light demonstrated by implementing back-end dielectric layers transparent to the UV, in combination with differential sensing circuits with polysilicon UV filters. Degradation of the developed sensors under short wavelength UV was investigated and design and operation regimes allowing decreased degradation were discussed. Compared with other embedded solutions, the current design is implemented in a mass-production CMOS SOI technology, without additional masks, and has high sensitivity in UVC.

## 1. Introduction

UV sensing has been widely researched in view of numerous applications, such as sterilization, flame monitoring, UV spectroscopy, UV cure processes, measuring of solar indexes, UV communications, and so on [1,2,3,4,5,6]. The target wavelength for these applications is in UVA (320 nm to 380 nm), UVB (280 nm to 320 nm), and UVC (200 nm to 280 nm) wavelength ranges.

Sterilization by UV radiation has been an extensively used technology in the past decades. UV radiation is very efficient in eliminating dangerous microbes in air and water and on different surfaces. The COVID-19 pandemic has sparked additional interest in using UV light for disinfection. It was reconfirmed by several groups that ultraviolet efficiently killed viruses, including SARS-CoV-2 [3,4,5,6]. UVB and UVC are destructive for COVID-19 RNA, while UVC with a wavelength shorter than 260 nm also damages the protein coats of COVID-19 viruses. The reported dose of UV radiation necessary for efficient disinfection is in the range of tens of mJ/(cm^2^) (or even 3.7 mJ/cm^2^ [3]), depending on the wavelength of UVB/UVC, properties of surfaces, and different environments [3,4,5]. Sterilization of COVID viruses strongly stimulated the production of germicidal mercury lamps, UVC LEDs, and various UV irradiating systems. Much of the recent innovation in the sterilization field has focused on devices for measuring parameters of UVC/UVB radiation integrated in irradiation setups. It is necessary to guarantee that the processed surfaces receive sufficient doses for sterilization. UV sensors are also needed to control the safety of people in the rooms, cars, airplane cabins, and other places where irradiation is performed. To guarantee the efficiency of sanitizing, it is necessary to measure the dose of ultraviolet in specific locations and in the presence of intense visible and IR illumination. Smart UV monitoring suggests large numbers of such sensors. Often, it is desirable to use them as elements of wireless sensor networks. Low-cost integrated solutions comprising sensors and low power communication enablement, such as RFID and Wi-Fi connectivity of the sensing nodes, are required for such systems. There are several solutions featuring UV sensors embedded into CMOS [7,8,9]; these technologies assume additional masks to the core CMOS process flow and typically have low responsivity in the UVC/UVB range.

Lateral PIN diodes in a thin silicon of SOI wafers were studied as candidates for UV sensors in [10,11,12,13]. For example, ML8511 UV sensors (LAPIS Semiconductor Co., Ltd., Yokohama, Japan) based on PIN photodiodes in thin silicon layer and supplied with an analog output were developed by OKI/LAPIS. These sensors detect 280–360 nm light and were shown to be efficient for controlling the intensity of “tanning rays” (UVA/UVB) in the solar spectrum. The sensitivity of the mentioned OKI sensor strongly decreases in the UVC range.

Connecting the lateral PIN SOI diodes in series for increasing the photovoltage was suggested in [12]. This solution was optimized to enable the design of UVC sensing devices. In this paper, we report on the performance of lateral PIN diodes integrated into the high-volume production RF SOI CMOS platform and designs that allow to increase the sensitivity to UVC. Special attention is paid to the SOI UV sensors’ degradation after irradiation with high UV doses and the way to decrease the influence of degradation. The main distinguishing features of the developed technology are the use of a high voltage string of PIN SOI diodes, UVC transparent CMOS back-end, and using non-salicided Polysilicon layer of standard CMOS as UV filter in differential approach, thus allowing high response to short wave UV compared with other technologies.

## 2. Materials and Methods

### 2.1. RF SOI CMOS Platform with Integrated UV Sensors

The modern RF CMOS platforms employ fully depleted (FD) or partially depleted (PD) SOI on high resistance silicon substrates. The very thin silicon channel layer and buried oxide (BOX) improve the ability to control the performance of MOS transistors, in particular allowing low junction capacitances and drain leakages, thus enabling RF functionality with reduced power consumption.

**Figure 1:**
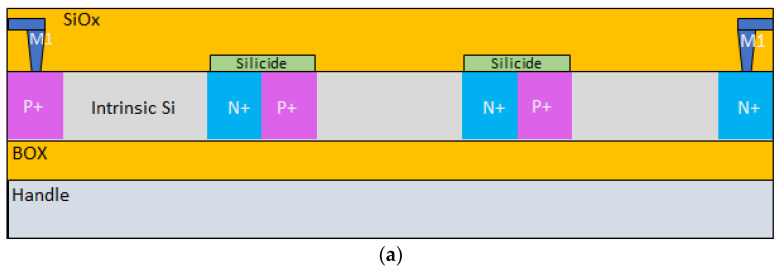

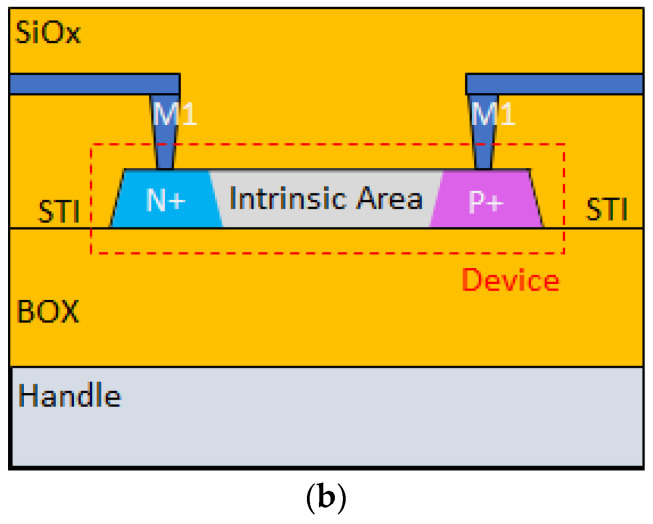
(**a**) A string of PIN photodiodes connected in series by silicide N+, P+, and I regions (Li). The diodes are connected by butted silicide. The schematical cross section shows only three connected in series PIN diodes. (**b**) Cross section of a lateral PIN diode with contacts.

Thin, 50–150 nm thickness, device layers of RF SOI are also suitable for making UV sensors. The devices presented in this paper were fabricated using mass production Tower Semiconductor 0.18 µm SOI process flow. No special diffusions (additional masks) were used. N+ and P+ source/drain implants penetrated to the bottom surface of SOI layers and defined the lateral PIN diodes. The “intrinsic” region corresponds to slightly p-type doped silicon of the SOI device layer (~3 × 10^15^ cm^−3^). The distinguishing feature of the developed sensors is the connection of diodes in series by silicide buts or by contacts at the M1 level. The connection of substate (special contact through BOX) is also a feature of the developed technology. A sensor with diodes connected by silicide [12] is shown in Figure 1.

The test structures used for UV sensors optimization included connected in parallel single PIN diodes. The test structures have the same total area (Table 1).

Most of the reported results are for devices where the silicon layer is ~1000 Å in the end of the process flow (partially depleted SOI). For physical models’ verification purposes, SOI wafers with single diodes formed in 0.6 µm SOI device layers and devices on bulk silicon were also investigated.

The back end of RF SOI CMOS that included UV sensors was modified to exclude UV absorbing layers and interference effects. A special type of passivation transparent to UVC was developed for these purposes. The total thickness of the optical window for UV including the passivation layer was 3 µm.

### 2.2. Test Setups

UV monochromators with calibrated spectral power densities were found to not be convenient for wafer-level characterization, when electrical contacts to a large number of light-sensitive test structures must be created, parasitic reflections must be avoided, and uniformity of illumination with different intensities must be guaranteed. As an alternative, a test setup that utilizes several UV and VIS light emitting diodes (LEDs) was built and shown to be efficient for engineering of the suggested UV sensors. We used four different UV LEDs: 255 nm (UV-C), 280 nm (UV-C/B), 310 nm (UV-B), and 365 nm (UV-A). Measurements in the visible wavelength range were also performed for comparison. LEDs, as light sources, were also utilized.

**Figure 2:**
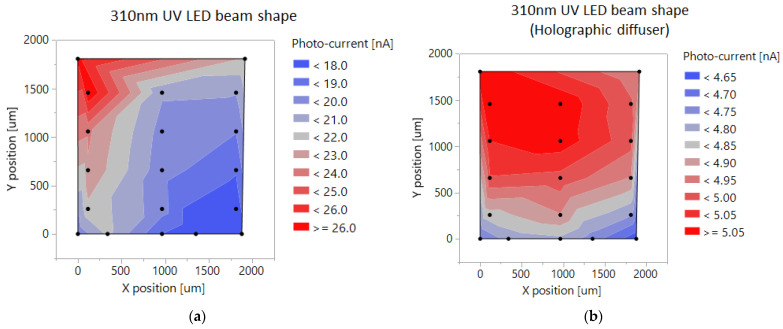
(**a**) A 310 nm LED beam without diffusers, photocurrent measured on a square 19 photo-diode matrix, with dimensions of 2 × 2 mm. (**b**) A 310 nm LED beam with holographic diffuser, photocurrent measured on the same photo-diode matrix.

The employed LEDs produce a non-uniform light, with the intensity having approximately Gaussian spatial distribution at the wafer surface. To reduce the non-uniformity, LEDs were positioned at 20–30 mm from the surface and devices placed were close to the maximum of the Gaussian distribution. To further improve the uniformity, a fused silica UV holographic diffuser from Edmund Optics (Barrington, NJ, USA) was used. This diffuser has over 85% transparency at 200–1500 nm wavelengths. The use of a diffuser helped to significantly improve the illumination uniformity, as illustrated in Figure 2a,b.

LEDs and diffusers were mounted on a 3D-printed base located above the tested chips. The base assures identical position of the LEDs with respect to the chips in different experiments, and thus high repeatability of the results.

Finally, for calibration of the LED optical power and calculations of sensor parameters, all the LEDs (paired with diffusers) were measured by a Newport 818-UV/DB + 843-R, calibrated detector + power meter combo [MKS Instruments Inc., Newport Corporation, Irvine, CA, USA]. Luminous intensity was calibrated for each wavelength used in the experiments.

This allowed to achieve the uniformity of irradiation and power density accuracy at different wavelengths at the level of 5–15%. The photocurrent was measured at $V_{D}=0\;V$.

Responsivity (R) [13] of devices was calculated, as in Equation (1):

(1) $$Responsivity=\frac{\left(\frac{I_{D}}{A_{D}}\right)}{P_{opt}}\;\left[A/W\right]$$

Here, the total diode area, $A_{D}$; LED optic power, $P_{opt}$; and measured current density, $I_{D}$, are used.

Measurements of photocurrent were performed with an HP4156C semiconductor analyzer. The specimens were placed on the chuck of the Cascade Microtech probe station. Custom designed boards, intended for UVC sensor performance demonstration, were also used in some of the experiments.

## 3. Results and Discussion

### 3.1. Embedded Lateral PIN Photodiode Performance

The parameters that influence photodiode performance, and that are easy to use for optimization without changing the process flow, are the following dimensions: $L_{i}$ (intrinsic area length), $W_{i}$ (intrinsic area width), $L_{n}$, $L_{p}$ (length of N+ and P+ terminals), and the back gate bias.

**Figure 3:**
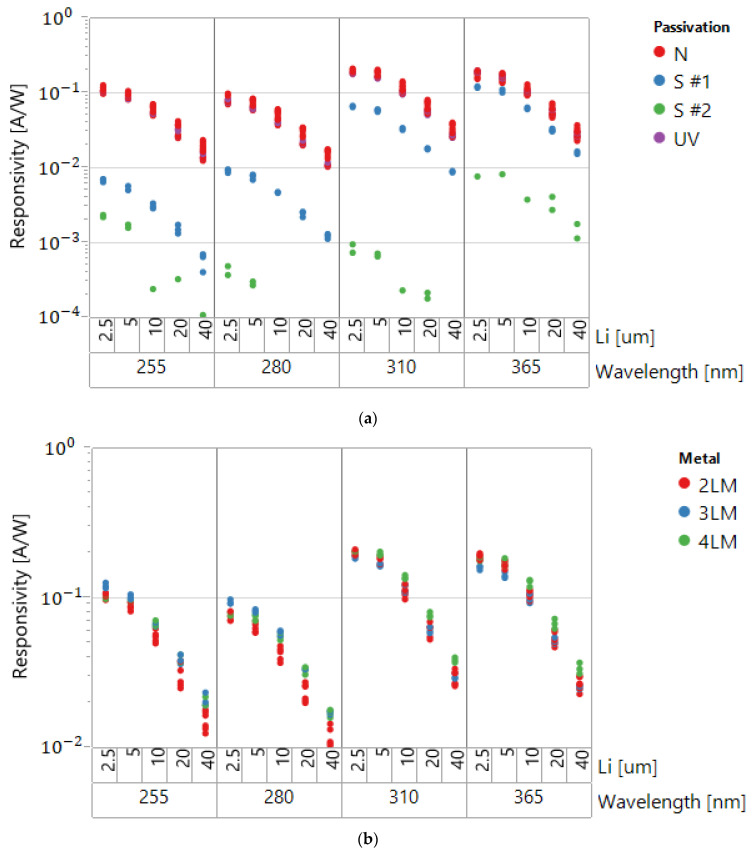
(**a**) UV sensors’ responsivity measured at four UV wavelengths for different Li and various back-end schemes, two levels of metallization, and three sites. (**b**) Responsivity for wafers without passivation, for different levels of metallization, and three sites.

The graphs in Figure 3a present the PIN diode responsivity as a function of $L_{i}$ in SOI with a 150 nm device layer and different types of the back end. The dots for each $L_{i}$ represent the data in three sites at the 200 mm wafer. Excellent repeatability is evident. The total device area was practically constant when changing $L_{i}$, as shown in Table 1.

The presented results are for S (standard flow not specially optimized for UV sensor embedding), N (the flow without passivation), and UV (the flow with special UV transparent passivation). All of the results were measured on single diodes connected in parallel. The results show that the standard passivation absorbs over 90% of UVC at 255 nm. The UV absorption decreases at longer wavelengths. “No passivation” (N) and specially engineered UV transparent passivation showed much better UV transparency, similar between the two splits. The number of metal layers (2LM–4LM, Figure 3b) had minimal impact on UV absorption.

At $L_{i}=2.5\;\mu m$, the values of R for sensors with optimized back-end reach ~0.2 A/W at 310–360 nm and 0.1 A/W at 255 nm, which is several times higher than in devices reported in [11,14]. The differences in sensitivity at different wavelengths, and specifically the decrease in sensitivity at 255 nm compared with 280 nm, are attributed to the reflectivity dependency on the wavelength of the UV light. Special antireflective coatings for UVC range, engineered for sterilization applications, can further increase the responsivity.

The R values start to saturate as the intrinsic length in the PIN diode decreases. It is clear that $L_{i}$ cannot be too small, because a sufficient sensing area is needed to absorb the radiation, while N+ and P+ regions of the sensors do not contribute to the photocurrent because of the generated electrons and holes recombination. At the same time, a larger $L_{i}$, above the values of ambipolar diffusion length $L_{D}$ of the generated in SOI layer electron–hole (e–h) pairs, do not add to the photocurrent [14]. The most sensitive devices ($L_{i}=2.5\;\mu m$) were selected as elements of the designed UV sensor strings of Figure 1.

**Figure 4:**
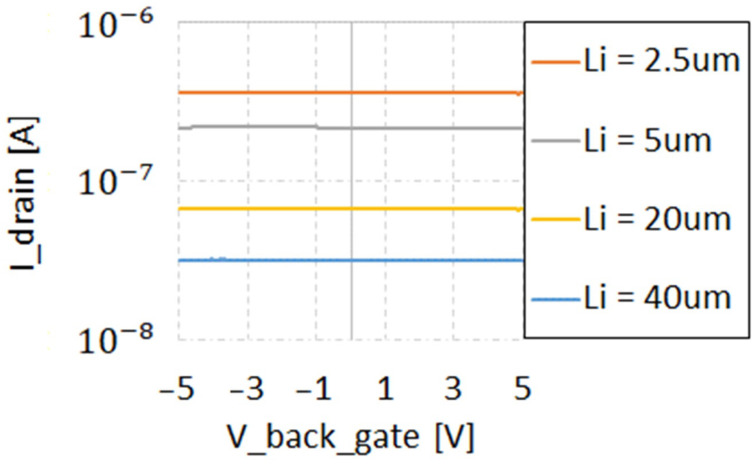
No dependence of fresh PIN detector photocurrent on the back bias. Here, 310 nm UV illumination is shown, with different Li.

Figure 4 shows the photocurrent as a function of the back-gate bias $V_{BG}$. The presented data are for illumination with 310 nm LED. At other wavelengths, practically no dependence on $V_{BG}$ was also observed.

This is different from the results reported in [10,11], where pronounced $V_{BG}$ dependence was reported. As mentioned above, the response of the PIN detectors depends on the ambipolar diffusion length of charge carriers before they reach the depleted regions, where there is a lateral electrical field separating e–h pairs. If $L_{i}$ is smaller than the diffusion length, only photons absorbed in the region with a parallel (to the Si surface) electrical field contribute to the photocurrent. If $L_{i}$ is larger than the depleted region (about 0.5 µm for the slightly P-type doped “intrinsic” region of the reported PIN diodes), then diffusion of charge carriers in the quasi-neutral or depleted by the vertical field $L_{i}$ region must be considered. With back gate bias, the surfaces in the $L_{i}$ region of the SOI device layer have “field-induced doping” [15]. For negative $V_{BG}$, induced P-type doping is connected to the P+ electrode of the PIN diode. For positive $V_{BG}$, an inversion layer is formed at the bottom surface of SOI device layer, so that the “field induced doping” region is connected to N+.

In the case of FD and PD SOI, the generated nonequilibrium electrons and holes are confined in the thin SOI device layer. In the case of vertical field, they continue to move laterally, bound together by Coulombic attraction [16]. For large enough $L_{i}$, for both back gate polarities, the region where separation of the diffusing electron–hole pairs happens is the lateral P–N+ junction. The holes (for negative $V_{BG}$) and electrons (for positive $V_{BG}$) exchange with accumulation and inversion “field-induced doping” regions. If the mobility in this region is the same as in the bulk, the ambipolar diffusion length is not affected for both negative and positive $V_{BG}$ polarities. This explains the observed weak dependence of photo-response on $V_{BG}$. On the contrary, with surface effects pronounced in SOI structures, the ambipolar diffusion coefficient is expected to be a function of the following: (i) the surface recombination at the bottom and/or top interfaces and (ii) increased scattering of charge carriers in “field-induced doping” regions. This can explain the differences in the reported results from [10,11], where lower responsivities and dependence of responsivity on the back-gate voltage were observed for SOI UV sensors. We argue that no dependence on the $V_{BG}$ relates to better quality of the starting SOI material compared with [10,11].

### 3.2. Degradation after High Doses of UV Radiation

**Figure 5:**
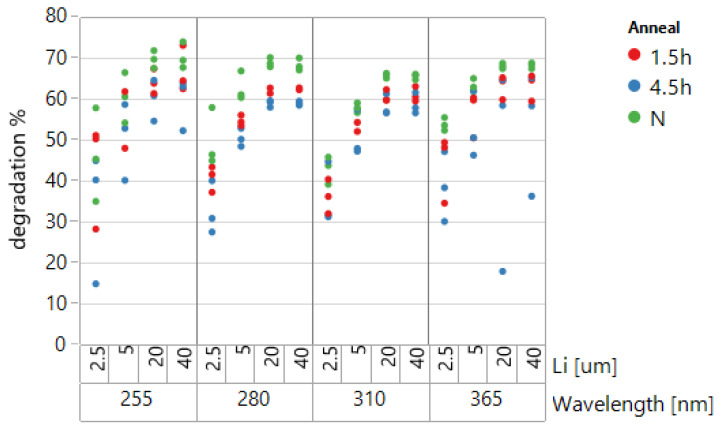
Degradation of thin SOI PIN diodes (with different intrinsic lengths), measured at four UV wavelengths before and after irradiation and annealed for 1.5 and 4.5 h (at 150 °C) (N-after 50 J/cm^2^ and before annealing). No passivation dielectrics. Three sites at the wafer.

Achieving high robustness to continuous UV exposure, with small degradation of responsivity over time, is one of the known challenges when using SOI diodes as UV sensors. Degradation, caused by the energetic UV radiation, results in traps’ generation at the silicon-silicon dioxide surfaces [17]. The fabricated PIN diodes were tested for degradation by exposing them to high UV doses. We used 254 nm radiation of C-91 EEPROM Eraser [UVP Memorase] with 4 mW/(cm^2^) intensity. Devices were measured before and after several cycles of exposure. The measurements of responsivity R included the influence of back gate voltage by the same methodology as in the previous sections: four calibrated LEDs: 255 nm, 280 nm, 310 nm, and 365 nm with holographic diffusers for uniform irradiation. After several exposure and measurement cycles, annealing was performed (several hours at 150 °C) to find whether the degradation of devices could be cured. The results for thin SOI diodes with different $L_{i}$ and for a 50 J/(cm^2^) UVC irradiation dose are presented in Figure 5. This dose is enough for thousands of COVID-19 sterilization cycles [3]. Degradation was calculated as the decrease in PIN unbiased diode photo current in % compared with the photo currents before the degrading exposure.

**Figure 6:**
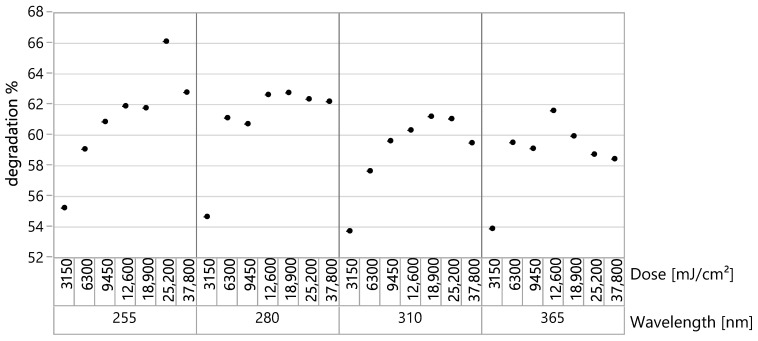
Thin SOI diode (Li = 20 µm) degradation after various doses of 254 nm UV.

After the long UVC irradiation, 40–70% degradation was observed. Degradation was more pronounced for large $L_{i}$ and saturated after irradiation doses of about 40 mJ/(cm^2^). There was a significant spread of currents for identical devices at the same wafer irradiated by large UVC doses. The spread reached tens of percent. The dependence of degradation on the irradiation dose is shown in Figure 6 for *L_i_* = 20 μm and different wavelengths of the registered UV.

The fact that sensors with large $L_{i}$ degraded significantly stronger suggests that the mechanisms responsible for ambipolar diffusion of e-h pairs are dominating. The degradation is attributed to trap generation at SiO_2_ interfaces. The traps facilitate recombination of generated by UV radiation electron–hole (e–h) pairs and result in mobility decrease of charge carriers, laterally diffusing to the regions of high lateral electrical field. The plausible mechanism of traps’ generation is desorption of hydrogen species in the employed silicon dioxide layers in contact with the SOI device layer.

The degradation of responsivity saturated with time. After high dose UV irradiation, it was still of the order of 0.1 A/W at 255 nm and stable in time. Thus, initial “curing” with intense UV radiation can be used in commercial solutions targeting very high doses.

**Figure 7:**
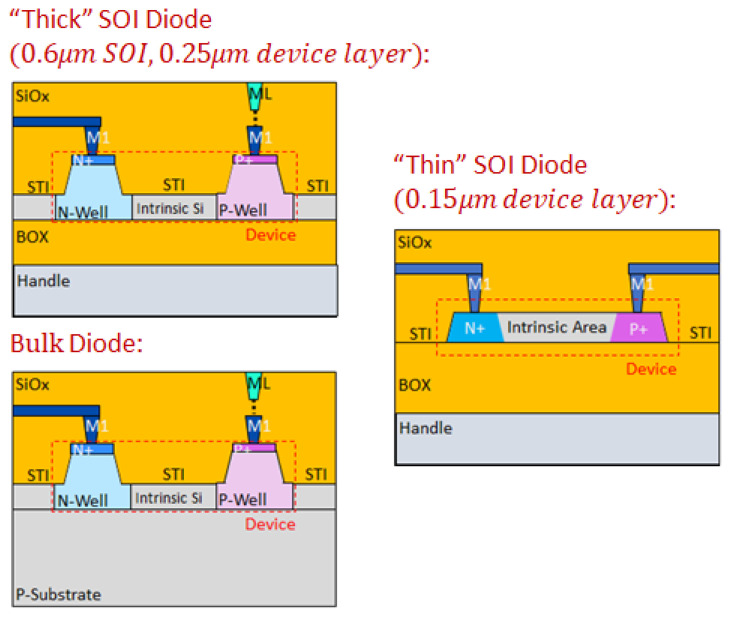
Schematic cross sections of thick, thin SOI, and bulk PIN diodes.

Special SOI PIN diodes having different device layer thicknesses and bulk diodes of similar geometry were used to distinguish between the degradation effects at the bottom and top surfaces of the SOI. The cross section of the designed devices is shown in Figure 7. “Thick” SOI and bulk PIN diodes were used in the performed studies besides the “thin” (~1000 Å final thickness device layer) SOI sensors. “Thick” SOI and bulk PIN diodes are similar, apart from the substrate below the device layer. The “thick” device layer on SiO_2_ (under STI) was ~0.25 µm. The SOI wafer technology for “thick” and “thin” SOI and doping of the device layer were the same. Thus, the properties of the surface facing the BOX of SOI were identical in both cases. In contrast with “thin” SOI devices, the 0.3 µm shallow trench isolation (STI) did not reach the BOX in “thick” SOI devices.

**Figure 8:**
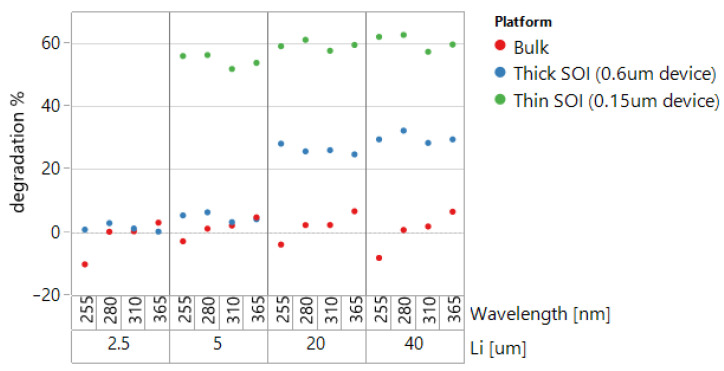
Thin/thick SOI and bulk PIN diode degradation with 254 nm UV dose of 12 J/cm^2^.

The “thin” SOI PIN diode has gate oxide (GOX) on its top Si-SiO_2_ interface, while the other two devices have STI as the top oxide. The results of the degradation experiment for three diode types are shown in Figure 8.

Thin SOI diodes experienced the highest degradation (around 60%), while thick SOI diodes had a significantly smaller decrease in photocurrent, pronounced only for large $L_{i}$. Bulk diodes showed practically no degradation for the employed UVC irradiation doses. Assuming that GOX interface has the same or better degradation immunity compared with the interface with STI, the results indicate that degradation happens mainly at the interface of SOI with BOX. The effect is less pronounced in “thick” diodes compared with “thin” devices because of the intensive UVC absorption in the silicon layer. In the case of “thick” SOI, the degrading UV radiation was strongly absorbed before reaching the bottom interface.

**Figure 9:**
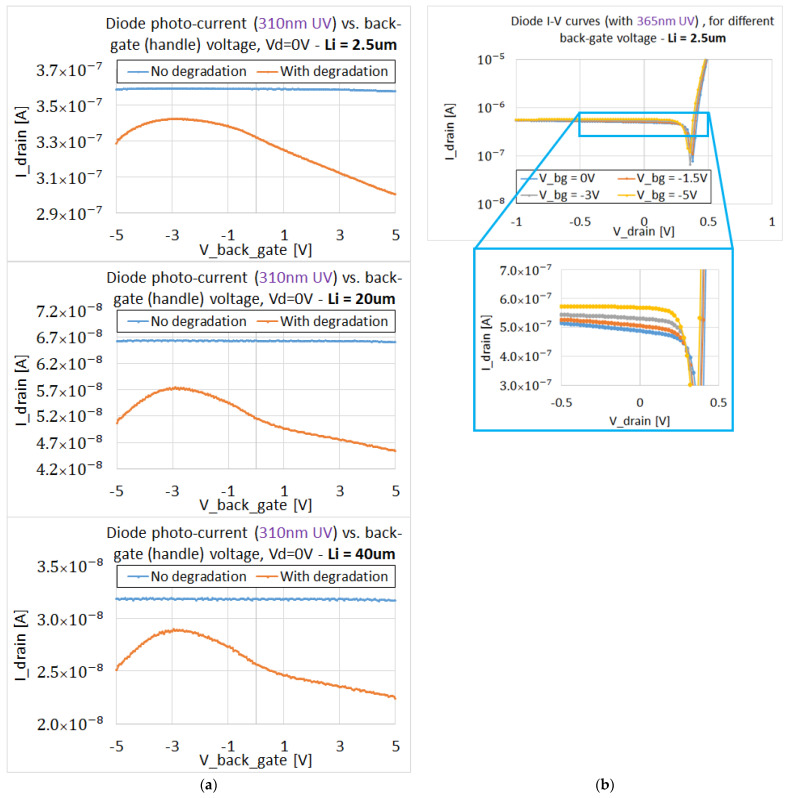
(**a**) Comparison of the back-bias voltage influence for degraded and fresh PIN SOI diodes, with different Li. (**b**) Typical Id-Vd curves (single PIN lateral diodes connected in parallel).

The experiments with back gate biasing (like in Figure 4) were also performed with the degraded devices. In this case, the obtained results were similar to those reported in [11,14]. The dependencies of the responsivity on the back-gate bias are shown in Figure 9. The decreased responsivity after high UVC doses depends on the back gate voltage $V_{BG}$. This dependence is more pronounced for $L_{i\;}>2.5\;\mu m$, when diffusion of charge carriers generated outside the depleted N+P—region of the PIN devices must be considered. R increases with negative $V_{BG}$ bias and then decreases for $\;|V_{BG\;}|>4\;V$. We argue that the increase in responsivity relates to the suppression of surface recombination (traps are filled with holes). For higher voltages, the decrease in R is attributed to the ambipolar mobility decrease. Within bulk models of ambipolar diffusion, this should be attributed to the change in electron mobility (as supposed in [11]). Nevertheless, in the case of e–h pairs’ diffusion in the confined space of thin SOI film, the influence of holes on the ambipolar diffusion coefficient cannot be neglected (imagine strong hole scattering by charged surface states). The decrease in responsivity at positive $V_{BG}$ could also be explained by mobility dependence.

The results presented in Figure 9a,b are for back gate voltage sweeps from −5 V to 5 V and drain voltage sweeps from −1 V to 1 V. Drain breakdown voltages of a single diode are greater than 10 V (not shown in Figure 9). With larger voltages applied to the back gate (up to +/−20 V), hysteresis effects were observed. This could be explained by trapping of holes and electrons at deep traps generated by large UVC doses, having time constants in the order of minutes. The hysteresis effects further confirm the generation of traps at SOI interfaces by high doses of UV radiation as the responsivity degradation mechanism.

From a practical viewpoint, PIN UV sensors on SOI must be engineered with minimum $L_{i}$ and minimum footprint of N+ and P+ regions, and operate with negative back gate voltage, in order to suppress the possible influence of the degradation effects.

### 3.3. UV Sensors with PIN Diode Strings

**Figure 10:**
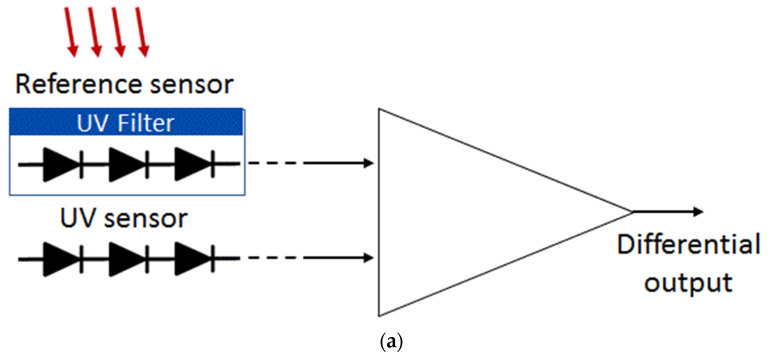

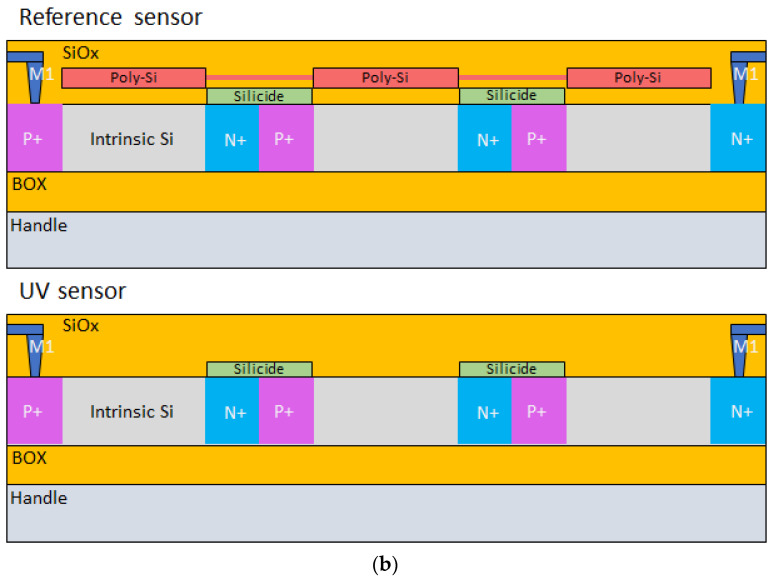
(**a**) A differential UV sensor comprising strings of UV sensitive lateral P–N junctions with and without UVC filters. (**b**) Cross section of a string of diodes with and without a UV filter.

With the employed 1000 Å SOI films, there is still a certain residual sensitivity to the UVA/UVB and visible/IR light components. This residual sensitivity becomes important when UVC intensity must be measured in the presence of visible light with large intensity. To improve UV spectral sensitivity, differential schemes in combination with SOI PIN sensors were considered and verified. We suggest a differential sensor comprising two PIN diode strings: one with UV blocking filters and one sensitive both to UV and visible light. By subtracting the signals of sensors, the response to visible light can be cancelled out. One of the designs is shown in Figure 10. The UV blocking layers are made of undoped polysilicon (Poly). This Poly (~2000 Å thick) is used in the core CMOS process as the material of MOS transistor gates and Poly resistors. An additional a-Si layer (500–1000 Å thickness) in the pre-metal dielectric of the standard CMOS process flow is one of the additional process options [18]. Spectral sensitivity can be tuned by changing the thicknesses of the mentioned UV blocking layers.

**Figure 11:**
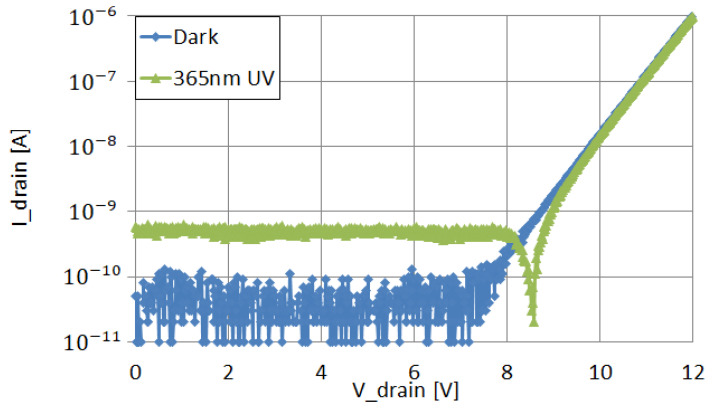
I–V characteristics of serially connected 17 thin SOI PIN diodes (by silicide), with and without illumination.

Serial connection of multiple diodes allows for larger voltage build up on the diodes when illuminated, as seen in Figure 11. $V_{drain}$ of 8.5 V corresponds to $V_{on}$ of 17 serial diodes.

**Figure 12:**
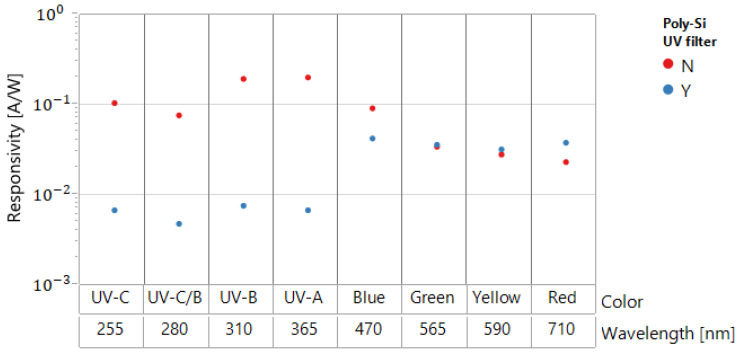
Photocurrent of thin SOI diodes (with different UV/visible wavelength for diodes having *L_i_* = 2.5 µm), with and without the Poly-Si UV filter.

**Figure 13:**
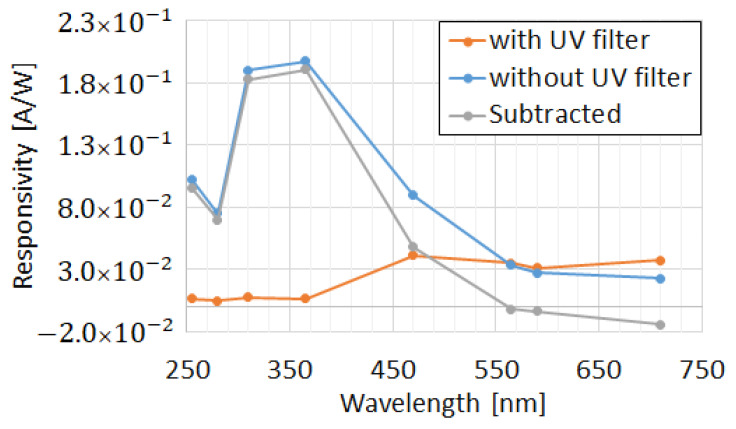
Thin SOI diodes’ (*L_i_* = 2.5 µm) spectral responsivity: improvement of visible spectrum “blindness” by connection of a “differential” pair of diodes.

The responsivity of diodes with (Y) and without (N) Poly filters is illustrated in Figure 12. Over 94% of UV light was absorbed by the Poly-Si UV filter, while the transparency for visible light (over 50% for blue light) increases with the wavelength. The transparency for red light was even better with Poly, possibly because of the interference effects connected with the polysilicon layer. The effectiveness of the differential device of Figure 10 is illustrated in Figure 13. In this case, UV is absorbed in the branch with the Poly filter, while the branch without the Poly filter reacts to both UV and residual visible and infrared light.

Strong suppression of the visible light is achieved while keeping high sensitivity in UVC and UVB (Figure 13).

The developed sensors can be used for both optical power sensing and dose calculation, if supplied with an integrator. Small size devices having a footprint of less than 1 mm^2^ are still practical. Their small signals for low power illumination sources are compensated in the suggested designs by connecting in series and thus generating high voltage. This voltage can be directly used in CMOS amplifying systems, as schematically shown in Figure 10.

## 4. Conclusions

Strings of PIN diodes connected in series with and without UV filters were integrated as elements of RF CMOS mass production process flow without additional masks. Large voltages generated by the strings under UV irradiation and availability of standard elements (MOS transistors, resistors, and capacitors) in the core CMOS platform allow to design UV sensing systems with a small footprint and low cost. Process flavors with a UV transparent back end enable a further increase in the UV responsivity. The single PIN diodes comprising the strings were optimized to obtain high responsibility and increased immunity to degradation after large UVC doses. Suggested differential designs subtracting the photocurrents generated by visible light allow to address applications when UVC light must be measured in environments with strong visible/IR radiation. The developed embedded technology allows designs where UV sensors and electronics for preliminary signal processing and RF communication are fabricated on the same SOI chip. To further increase sensitivity to UVC, investigations into the device flavors on FD SOI are in progress. The planned research will also include the influence of operation temperature on degradation performance.

## Figures and Tables

**Table 1:** Dimensions of diodes used in the experiment.

Device #	$L_{i}$ [μm]	$W_{i}$ [μm]	$N_{parallel}$	Intrinsic Area [μm^2^]	Total Area [μm^2^]
1	2.5	100	2233	558,250	687,918
2	5	100	1155	577,500	644,644
3	10	100	623	623,000	659,288
4	20	100	315	630,000	648,424
5	40	100	161	644,000	653,618

## Data Availability

Raw data related to the measurements of developed devices are available. Specimens of the fabricated UV sensors as well as demonstration kits can be provided to interested parties under individual agreements.

## Abbreviations

The following abbreviations are used in the manuscript:

CMOS	Complementary metal oxide semiconductor
UV	Ultra-violet
RF	Radio frequency
SOI	Silicon on Insulator
IR	Infra-red
LED	Light emitting diode
RFID	Radio frequency identification
VIS	Visible spectrum
FD	Fully depleted
PD	Partially depleted
MOS	Metal oxide semiconductor
BOX	Buried oxide
STI	Shallow trench isolation
PVD	Photo-voltaic diode
BG	Back-gate
GOX	Gate oxide
